# Ethnic-Racial Identity and Alcohol Use Moderated by Family Factors among Diverse Emerging Adults

**DOI:** 10.1007/s12552-025-09489-3

**Published:** 2026-01-24

**Authors:** Chloe J. Walker, Chelsea Derlan Williams, Arlenis Santana, Eryn N. DeLaney, Jamie Cage, Jinni Su, Sally I. Kuo, The Spit for Science Working Group, Danielle M. Dick

**Affiliations:** 1 University of North Carolina, Chapel Hill, North Carolina USA; 2 Virginia Commonwealth University, Richmond, Virginia USA; 3 Arizona State University, Tempe, Arizona USA; 4 Rutgers University, New Brunswick, New Jersey USA

**Keywords:** Ethnic-racial identity/ethnic identity/racial identity, Parent education, Family history of alcohol problems, Alcohol use, Emerging adults

## Abstract

Excessive alcohol use is associated with adverse outcomes, underscoring the importance of identifying factors that may reduce alcohol use among diverse emerging adults, including ethnic-racial identity (ERI) and family factors. Limited work has examined factors that moderate the relations between ERI and alcohol use. The current study tested whether family factors (i.e., parent education and family history of alcohol problems) moderated the relations between ERI and alcohol use among 1850 diverse college students, ages 18–22 (*M* = 18.46, *SD* = .38). Findings indicated that moderation effects varied by students’ ethnicity/race**.** At high levels of parent education, greater ERI resolution predicted less alcohol use among Asian individuals, and greater alcohol use among White individuals. Among Multiracial individuals with lower family history of alcohol problems, greater ERI exploration was related to less alcohol use. Findings highlight nuanced ways that ERI, parent education, family history of alcohol problems, and racial differences influence college students’ alcohol use. Results have implications for alcohol prevention and intervention programs by highlighting that both ERI development and family influences should be discussed by therapists, program leaders, and mentors with emerging adults across racial backgrounds.

## Introduction

A majority of full-time college students in the U.S. consumed alcohol in the past month (NIAAA, 2024), and harmful and underage drinking impairs the intellectual and social lives of students across campuses (National Institute on Alcohol Abuse and Alcoholism, 2014). Given the prevalence and outcomes associated with excessive alcohol use, existing research has identified factors, such as ethnic-racial identity (i.e., ERI), that play a role in reducing alcohol use (e.g., Bravo et al., 2025; Walker et al., 2022). ERI is a multidimensional construct consisting of process and content dimensions (Umaña-Taylor et al., 2014; Williams et al., 2020). Process dimensions involve the behaviors that individuals engage in to develop their ERI, such as exploration (i.e., searching and learning more about one’s ethnic-racial group) and resolution (i.e., gaining a sense of clarity about being a member of one’s ethnic-racial group). Content dimensions involve one’s attitudes and beliefs about their ethnic-racial group membership, such as affirmation (i.e., the positive or negative feelings one has toward being a member of their ethnic-racial group).

Extant work examining how ERI impacts alcohol use has predominantly focused on content dimensions of ERI, finding that greater ERI affirmation is associated with less alcohol use (Bowman Heads et al., 2018; Zapolski et al., 2017). However, we know less about the role of process dimensions (e.g., exploration and resolution) in alcohol use reduction. Understanding the processes involved in forming an identity is important, particularly during emerging adulthood (18–25 years old) within a college context (Williams et al., 2020). Furthermore, limited work has examined factors that may moderate the relations between ERI process dimensions and alcohol use. Therefore, to address these gaps, the current study examined whether family factors moderated the association between ERI exploration and resolution and alcohol use among diverse emerging adults.

## Consideration of Family Factors as Moderators

The Phenomenological Variant of Ecological Systems Theory (PVEST; Spencer, 2006) posits that in examining identity and outcomes, it is important to consider how structural, cultural, individual, social, and contextual factors play a role. Accordingly, given the importance of the family context, family factors that may moderate the relations between identity (e.g., ERI) and outcomes (e.g., excessive alcohol use) are parent education and family history of alcohol problems. While no studies have examined these family factors as moderators of these associations, previous work has indicated that family factors are directly related to alcohol use among emerging adults (e.g., Powers et al., 2017; Ross et al., 2025).

### Parent Education

Parents with higher education instill messages in their children about the importance of education as a way to uphold their family legacy and become successful (Augustine, 2017; Mortimer & Lee, 2021). It is possible that college students whose parents have higher education may feel increased pressure to represent their family and ethnic-racial group well. Thus, they might use alcohol excessively as a way to cope with achievement expectations and pressures from their parents while they navigate college and pursue their education. Consistent with this notion, prior work has suggested that young adults with high socioeconomic status (SES) tend to consume greater alcohol than young adults with lower SES (Collins, 2016; Martin, 2019) because they experience greater achievement pressure (Luthar, 2006). Therefore, it is possible that ERI will predict less alcohol use among students whose parents have lower parent education, and ERI may not predict alcohol use among students whose parents have higher education.

### Family History of Alcohol Problems

Family history of alcohol problems is associated with increased risk for alcohol use among young adults (Kendler et al., 2015; Ross et al., 2025). Even though engaging in ERI formation is a positive process during emerging adulthood (Elias et al., 2025; Williams et al., 2020), the increased risk associated with family history of alcohol problems may impede this process. Thus, ERI may inform less alcohol use but only among students with less family history of alcohol use (i.e., have fewer family members with alcohol problems). On the other hand, ERI may not be associated with less alcohol use among students who have a higher family history of alcohol problems (i.e., more family members with alcohol problems). Although no work has tested whether relations between ERI dimensions and alcohol use are moderated by family history of alcohol problems, prior work provides support that family history of alcohol negatively impacts engaging in normative, positive processes (similar to the normative, positive process of ERI formation). For example, findings from a study of college students found that those with higher family history of alcohol problems reported that they engaged less frequently in enjoyable, evening substance-free activities (Joyner et al., 2018). Further, prior work found that higher rates of maternal substance use exposure during childhood was associated with greater substance use in emerging adulthood (Ross et al., 2025).

## Current Study

Overall, alcohol use increases during emerging adulthood, making it especially important to test predictors of it during this period. Although some work has tested the role of family factors in alcohol use, no work has examined whether parental education and family history of alcohol moderate relations between ERI and alcohol use. To address gaps in existing literature, the current study explored whether family factors (i.e., parent education and family history of alcohol problems) moderated associations between ERI and alcohol use among diverse college students. We hypothesized that ERI would predict less alcohol use among students with less family history of alcohol problems and whose parents had lower education. We also hypothesized that these associations would not be significant among students with higher family history of alcohol problems and whose parents had higher education.

## Method

### Participants and Procedure

Participants in the present study were part of a larger, university-wide longitudinal study examining the predictors of college students’ substance use and emotional health outcomes (Dick et al., 2014)The larger sample includes five cohorts of college students at an urban, public university in the southeastern U.S. The present study focused on 1850 emerging adults from the larger study who identified as White (*n* = 814), Black/African American (*n* = 420), Hispanic/Latinx (*n* = 112), Asian (*n* = 385), and more than one race (*n* = 119). Data were from the Spring 2017 data collection because this was the first and only wave to include a measure of ERI. Participants were 18 to 22 years old (*M* = 18.46, *SD* =.38), with the majority identifying as female (i.e., 69%). Additionally, most students lived in a residence hall on campus (70%) and were not currently working (60%).

Each year, incoming freshmen were asked to participate in the larger study and complete a survey online after providing informed consent online. The survey took approximately 15–30 min to complete, and participants received $10 compensation. Study data were collected and managed using REDCap (Research Electronic Data Capture), which is a web-based application designed to assist with data capture for research studies (Harris et al., 2009).

## Measures

### ERI

The exploration and resolution subscales from the brief form of the Ethnic Identity Scale (EIS-B; Douglass & Umaña-Taylor, 2014) were used to assess ERI. The exploration subscale is 3 items (e.g., “I have attended events that have helped me learn about my ethnicity.”) and the resolution subscale is 3 items (e.g., “I know what my ethnicity means to me.”). Responses were rated on a 4-point Likert Scale, in which 1 = *Does not describe me at all*, and 4 = *Describes me very well*. Support for validity and reliability of the EIS-B among diverse individuals has been provided by previous work (Douglass & Umaña-Taylor, 2015; Umaña-Taylor et al., 2018). Alphas in the current study were .88 for exploration and .88 for resolution.

### Alcohol Use

Alcohol use was measured using one-item: “How often do you have a drink containing alcohol?”. Options ranged from 1 = *Never* to 5 = *Four or more times a week*.

### Family History of Alcohol Problems

Responses from four items in the survey were used to assess family history of alcohol problems (i.e., history for mother, father, aunts/uncles/grandparents, and siblings; Kendler et al., 2015). For example, students were asked: “Do you think your biological mother has ever had problems with alcohol? (By problems with alcohol we mean that her alcohol use caused problems at home, at work, with her health, or with the police, or that she received alcohol treatment).” The family factors score was created using a standardized mean of the responses for all four categories of relatives.

### Parent Education

Parent education was reported by students for up to two parents. For students who indicated two parental figures, the highest level of education of the two parental figures was used, and for students who indicated one parental figure, the level of education they reported was used.

## Analytic Approach

Three fit indices were used to examine model fit: the comparative fit index (CFI), the root-mean-square-error of approximation (RMSEA), and the standardized root-mean-square residual (SRMR). Model fit was considered to be good if the CFI was ≥.95, and the RMSEA and SRMR were ≤.05 (Hu & Bentler, 1999). To test hypotheses, we specified two models. The first model tested whether relations between ERI dimensions (i.e., ERI exploration and ERI resolution) and alcohol use were moderated by parent education. The second model tested whether relations between ERI dimensions (i.e., ERI exploration and ERI resolution) and alcohol use were moderated by family history of alcohol problems.

We also included cohort and gender as covariates. Given that prior research has indicated that there are ethnic-racial group differences in how ERI informs alcohol use (i.e., Zapoloski et al., 2017), we examined ethnic-racial group differences by specifying the two models as multigroup models that included ethnicity/race (i.e., Asian, African American, White, Latinx, and Multiracial) as the grouping variable. All predictor variables were mean-centered, and any significant moderators were probed at one standard deviation below the mean and one standard deviation above the mean (Preacher et al., 2006).

## Results

**Fig. 1:**
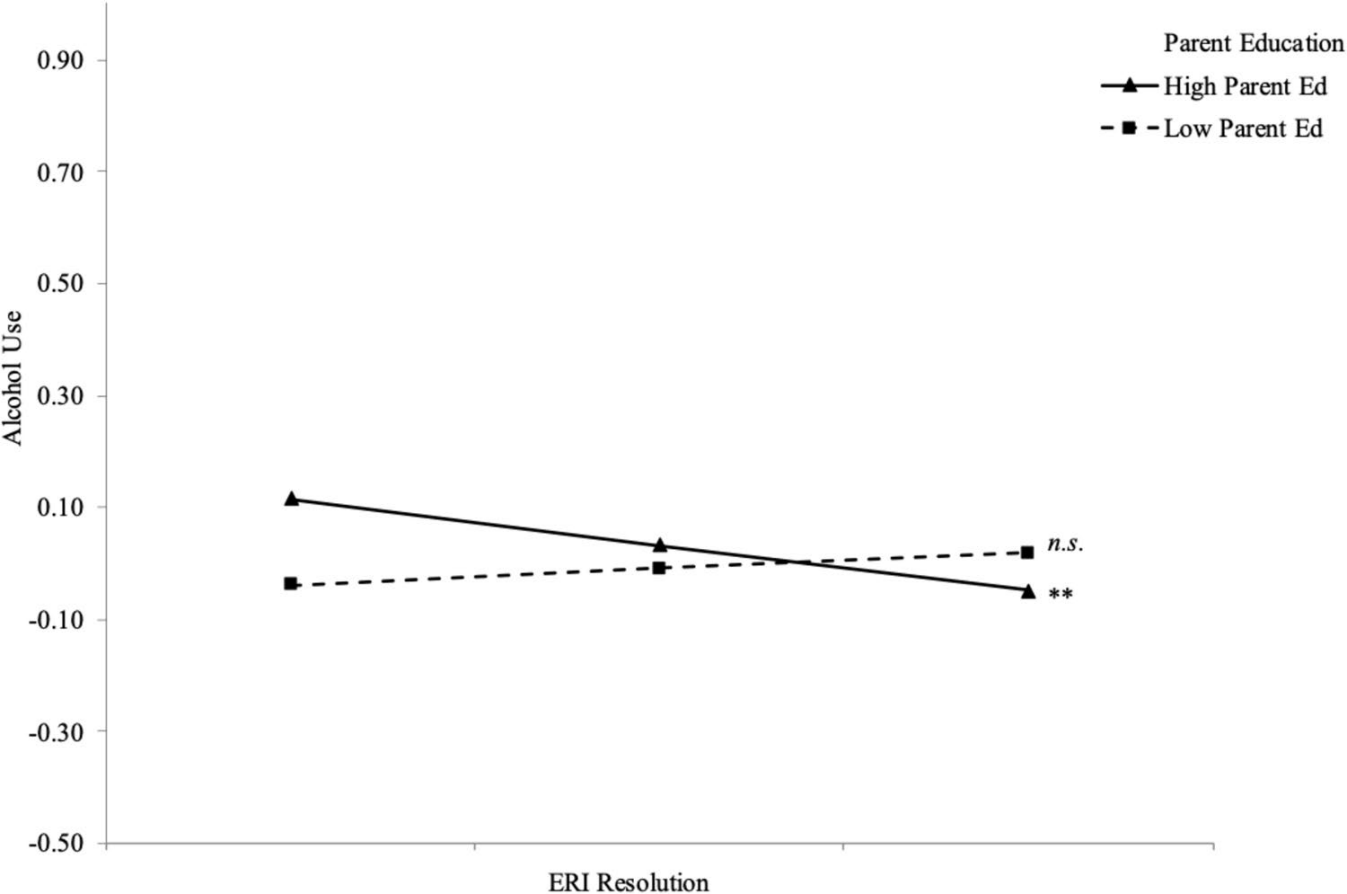
Moderation effects of parent education on the association between ERI Resolution and alcohol use among Asian college students (*n* = 385)

**Fig. 2:**
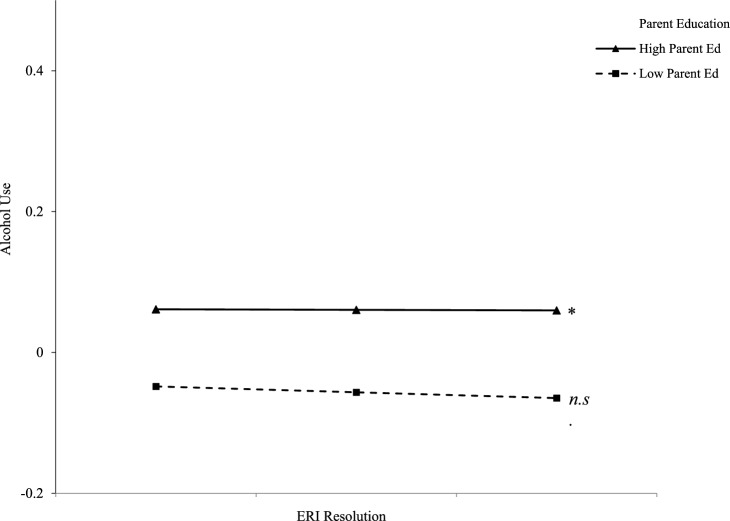
Moderation effects of parent education on the association between ERI Resolution and alcohol use among White college students (*n* = 814)

First, means, correlations and standard deviations were computed for all study variables (see Table 1). All analyses tested hypotheses in MPlus 7.31 (Muthen & Muthen, 2014) with full information maximum likelihood to handle missing data (Enders, 2013). The first model with parent education as a moderator of relations between ERI and alcohol use had good fit, *χ*^*2*^ (*df* = 10) = 30.37, *p* =.001, RMSEA =.07, 90% CI [.05,.10], CFI =.99, SRMR =.02 (see Table 2). Findings indicated that among Latinx students, greater ERI resolution predicted less alcohol use and individuals whose parents had more parent education predicted more alcohol use. For Multiracial students, greater ERI predicted greater alcohol use. Additionally, for White students, greater ERI exploration predicted less alcohol use. Regarding moderation, parent education was a significant moderator of the relation between ERI resolution and alcohol use among Asian students and White students. Simple slopes analyses indicated that among Asian students whose parents had high education, greater ERI resolution was significantly related to less alcohol use, and this relation was not significant among Asian students whose parents had low education (See Fig. 1). Among White students whose parents had high education, greater ERI resolution was significantly related to more alcohol use, and this relation was not significant among White students whose parents had low education (see Fig. 2).

**Table 1:** Means, Standard Deviations, and Correlations Among Study Variables (N = 1850)

	1	2	3	4	5	6	7
1. ERI Exp	–						
2. ERI Res	.86**	–					
3. Parent Education	.00	−.00	–				
4. FH of Alc Problems	.06*	.06*	−.04	–			
5. Alcohol Use	−.01	−.01	.08*	.13*	–		
6. Cohort	.05	.03	.06*	.14	.04	–	
7. Gender	−.02	−.04	.04	.06*	-.12*	.06*	–
Mean	2.23	3.01	7.64	.01	2.88	3.36	1.69
Standard Deviation	1.00	.87	1.71	2.33	.99	.72	.46

*ERI* ethnic-racial identity, *Exp* exploration, *Res* resolution, *FH* family history, *Alc* alcohol, Gender was coded, female = 1 and male = 0

^*^p ≤.05. ** *p* ≤.01. *** *p* ≤.001

**Table 2:** Final path analysis of the associations between ethnic-racial identity and alcohol use moderated by parent education among Asian (*n* = 385), Black (*n* = 420), Latinx (*n* = 112), Multiracial (*n* = 119), and White (*n* = 814) College Students

Path	Estimate (*SE*)				
	Asian	Black	Latinx	Multiracial	White
ERI Exp → Alcohol use	.10 (.10)	.06 (.06)	.12 (.12)	−.20 (−.19)	−**.10 (**−.09)*
ERI Res → Alcohol use	−.14 (−.11)	.01 (0.01)	−.33 (.23)*	.28 (.24)*	.04(.04)
Parent education → Alcohol use	.01 (.02)	−.03 (−.06)	.09 (.21)*	.03 (.04)	.03 (.04)
ERI Exp x Parent education → Alcohol use	.04 (.10)	−.00 (−.01)	.03 (.07)	−.07 (−11)	−.04(−.04)
ERI Res x Parent education → Alcohol use	−.15 (−.22)***	.03 (.05)	.04 (.06)	−.04 (−.05)	.06 (.08)*

*ERI* ethnic-racial identity, *Exp* exploration, *Res* resolution, Unstandardized path estimates are displayed. Standardized path estimates are displayed in parentheses. Significant paths are bolded. * *p* <.05. ** *p* <.01. *** *p* <.001

Regarding the second model with family history of alcohol problems as a moderator of the relations between ERI and alcohol use, the model had good fit, *χ*^*2*^ (*df* = 10) = 25.85, *p* =.003, RMSEA =.07, 90% CI [.04,.10], CFI =.98, SRMR =.02 (see Table 3). Findings indicated that for White students, greater ERI exploration predicted less alcohol use. Additionally, among White students, having more family members with a history of alcohol problems predicted greater alcohol use. Regarding moderation, findings indicated that family history of alcohol problems was a significant moderator of the relation between ERI exploration and alcohol use among Multiracial students. Specifically, simple slopes analysis indicated that at low family history of alcohol problems (i.e., fewer family members with a history of alcohol problems), greater ERI exploration was significantly related to less alcohol use, but this relation was not significant among Multiracial students with higher family history of alcohol problems (see Fig. 3).

**Table 3:** Final path analysis of the associations between ethnic-racial identity and alcohol use moderated by family history of alcohol problems among Asian (*n* = 385), Black (*n* = 420), Latinx (*n* = 112), Multiracial (*n* = 119), and White (*n* = 814) College Students

Path	Estimate (*SE*)				
	Asian	Black	Latinx	Multiracial	White
ERI Exp → Alcohol use	.08 (.08)	.08 (.08)	.21 (.20)	−.15(−.15)	−.11 (−.10)*
ERI Res → Alcohol use	−.14 (−.11)	−.01 (−.00)	−.77 (−.54)	.16 (.14)	.06(.05)
FH of Alcohol problems → Alcohol use	−.17 (−.05)	−.05 (−.02)	.81 (.28)	−.41 (−.13)	.33 (.12)*
ERI Exp x FH of Alc problems → Alcohol use	−.80 (−.20)	−.45 (−.17)	.52 (.19)	1.20 (.38)**	−.25(−.07)
ERI Res x FH of Alc problems → Alcohol use	.34 (.08)	.29 (.07)	−1.43 (−.32)	−.92(−.28)	−.01(−.00)

*ERI* ethnic-racial identity, *Exp* exploration, *Res* resolution, *FH* family history, *Alc* alcohol, Unstandardized path estimates are displayed. Standardized path estimates are displayed in parentheses. Significant paths are bolded. * *p* <.05. ** *p* <.01. *** *p* <.001

## Discussion

Based on PVEST (Spencer, 2006), the current study tested whether ERI would be associated with alcohol use, and whether these relations would be moderated by parent education and/or family history of alcohol problems. We hypothesized that (1) ERI would be associated with less alcohol use among students with less family history of alcohol problems and whose parents with lower education and, (2) these relations would not be significant among students with higher family history of alcohol problems and whose parents had higher education. Expectations were not supported for parent education, but our hypothesis was supported for family history of alcohol problems, yet findings varied based on students’ ethnic-racial background.

### Parent Education

Results indicated that among Asian and White students, parent education moderated the relation between ERI resolution and alcohol use, but in opposite directions. Specifically, among Asian students, ERI resolution significantly predicted less alcohol use at high parent education. Asian students whose parents have higher education may have received messages to continue their family’s legacy of education by representing their family in a positive way as they navigate college. In fact, research has shown that some Asian individuals tend to fulfill their filial piety obligation (a cultural principle of showing respect and love toward their parents and ancestors) to their parents through their academic achievements in school (Chow & Chu, 2007). Based on this notion, Asian students whose parents have higher education may want to maintain that positive perception of themselves, their family, and their ethnic-racial group, and so as they gain more resolution about what it means to be part of their ethnic-racial group, they participate less in alcohol-related activities like alcohol use. However, given that no work has been conducted with parent education as a moderator of ERI and alcohol use, more research is needed that addresses these notions about legacy messaging in the context of ERI development more directly.

Interestingly, ERI resolution was associated with *more* alcohol use among White students whose parents had higher education. This finding may reflect the differences in how White students think about race and form their ERI in college, compared to Asian students. Prior work suggests that White individuals with higher ERI centrality (i.e., how important and central one’s race is to their sense of self) are more likely to feel responsible and even guilty for the historical wrongdoings of their ethnic-racial group (Knowles & Peng, 2005).

White students may experience more racial engagement and consideration of what it means to be White with privilege and/or in the majority group for the first time in college. It could be that resolving what it means to be White among individuals with highly educated family backgrounds brings awareness to the multiple forms of privilege of White students with highly educated families. Additionally, the sociopolitical context may have heightened this awareness for White students, given that the present study occurred after the 2016 election that focused on race throughout the election and afterward (Sides et al., 2018). Recent research suggests that White individuals have become increasingly anxious about their groups’ status and position in society (Berry et al., 2019; Mutz, 2018). Given that ERI resolution was occurring within a university context that is composed of 45% ethnic-racial minority students in a sociopolitical environment that has shown hostility toward minorities, White students in the current study may have felt increased guilt and negative feelings about their multiple privileges that they may have coped with by increasing alcohol use. However, more work is warranted that tests ERI, family factors, and alcohol use in additional sociopolitical and university contexts.

### Family History of Alcohol Problems

Family history of alcohol problems was a significant moderator of relations between ERI exploration and alcohol use among Multiracial students. Consistent with expectations, among Multiracial students with low family history of alcohol problems, greater ERI exploration was linked with less alcohol use. Prior work examining the role of ERI exploration and alcohol use among emerging adults is scant. However, work that has been conducted in this area with Multiracial youth has found that ERI is protective against alcohol use (e.g., Choi et al., 2006, Fisher et al., 2017). Multiracial individuals have unique experiences with forming their ERI, such that they may think about their races/ethnicities differently than monoracial individuals (i.e., individuals with only one ethnic-racial background, such as Asian), which may explain why findings only emerged for them. ERI exploration may be particularly protective for Multiracial students because it helps them learn more about their backgrounds and gain a sense of who they are, leading to less alcohol use when they do not have a family history of alcohol problems. Overall, given that this is the first study to our knowledge to test the moderating effects of family history of alcohol problems in these associations among Multiracial college students, more research is needed that clarifies the mechanisms underlying why these relations emerged.

## Limitations, Implications, and Conclusions

There are various limitations to acknowledge. First, this study was cross-sectional; thus, casual relations cannot be assumed. Second, future studies should test whether different findings emerge if other indices of SES besides parent education (e.g., students’ income, financial support, parent employment status) are included as moderators. Additionally, findings indicated meaningful differences among Asian, White, Multiracial, Latinx and African American students; however, due to the small number of other underrepresented ethnic-racial minority students (e.g., Native American students), we were unable to test for significant differences among other groups. It will be important for future work to recruit larger samples to be able to test these differences. Although surveys were de-identified, confidential, and completed online, it is still possible that students underreported their alcohol use due to social desirability and due to some of the sample being underage. Future work is needed with larger samples of emerging adults over 21 to assess whether different findings emerge. Lastly, to reduce participant burden in the overall study, alcohol use was assessed with one item. Our findings provided important foundational information regarding the factors underlying use, but future work is warranted that builds upon this study using more nuanced assessments.

Despite these limitations, the present study has various implications. Regarding research implications, our findings highlight that culture should be assessed in nuanced ways (e.g., testing ethnic-racial background differences, including ERI assessments) when focusing on alcohol use. These relations are complex, and must be considered in a multitude of ways in order to fully capture the experiences of emerging adults and reduce alcohol. In terms of clinical and practice implications, these findings may be useful for alcohol prevention and intervention programs by suggesting that both ERI development and family influences should be discussed by therapists, program leaders, and mentors aiming to target alcohol use.

Overall, the current study provides insight into the way that family factors and ethnicity/race influence the relations between ERI dimensions and alcohol use among diverse emerging adults in college. Findings highlight that considering students’ ERI and familial contextual experiences are important in understanding the complexity of factors influencing alcohol use.

## Data Availability

Data from this study are available to qualified researchers via dbGaP (phs001754.v4.p2) or via spit4science@vcu.edu to qualified researchers who provide the appropriate signed data use agreement.
